# Commentary: Proceedings of the First Workshop on Peripheral Machine Interfaces: Going beyond Traditional Surface Electromyography

**DOI:** 10.3389/fnbot.2017.00032

**Published:** 2017-07-05

**Authors:** Philipp Beckerle

**Affiliations:** ^1^Institute for Mechatronic Systems, Mechanical Engineering, Technische Universität Darmstadt, Darmstadt, Germany

**Keywords:** human–machine interfaces, human factors, human-oriented design, prosthetics, rehabilitation robotics

Peripheral machine interfaces are an important field of exoprosthetic research since they facilitate the communication between human and robotic device and thus their collaboration. Castellini et al. (2014) give a very well-researched overview of the users’ demand for better control and the limiting factors in recent academic and commercial approaches. The paper is currently among the top 10 viewed papers in Frontiers in Neurorobotics and focuses on upper limb robotic prostheses. This commentary contemplates the review of Castellini et al. (2014) from a human-oriented perspective and regarding the lower limbs. Beyond the reported technical challenges in mechatronic and control design (Peerdeman et al., 2011; Castellini et al., 2014), the consideration of human factors such as acceptance and especially embodiment of the devices seems to be of central importance (Giummarra et al., 2008; Christ et al., 2012; Beckerle et al., 2017a). Castellini et al. themselves emphasize the tight relation of human factors and technical approaches by tracing lacks of device embodiment to limited control performance, reduced prosthetic dexterity, and missing afferent feedback (Castellini et al., 2014). While this connection to human demands further agrees with current research on personalized neuroprosthetics (Borton et al., 2013), this commentary focuses on non-invasive interfaces in accordance with Castellini et al. (2014).

From the users’ perspective, predictability of motion behavior and transparency of control appear to be crucial for embodiment irrespective of the considered extremity (Castellini et al., 2014; Veneman et al., 2017). Castellini et al. (2014) point out that current electromyographic approaches to control multi-fingered prosthetic hands are non-physiological and non-intuitive. Hence, they are not only cognitively burdensome for their users but furthermore seem to subconsciously counteract embodiment. Increased cognitive effort might even be related to reduced embodiment going along with frustration (Castellini et al., 2014; Makin et al., 2017). Moreover, both, increased cognitive effort and reduced embodiment, affect user satisfaction which suits the observation of frequent device abandonment (Jiang et al., 2012). Castellini et al. argue that advanced algorithms predicting the user’s intention shall improve functional outcome and satisfaction through faster control. Remarkably, similar correlations between biomechanical functionality, user satisfaction, and human–machine interfaces are found in lower limb prosthetics (Beckerle et al., 2017a). Transparency of control is a paramount objective in upper and lower limb prosthetics but hard to reach due to the motion complexity and human–machine interface limitations such as low bandwidth and missing sensory feedback (Makin et al., 2017; Veneman et al., 2017).

One of the technical suggestions of Castellini et al. is the investigation of semi-autonomous systems. While this is promising due to decreasing cognitive effort, such devices need to be carefully designed since increased autonomy might reduce the users’ experience of agency, i.e., the feeling to be able to control the device, and thereby embodiment. Correspondingly, some users prefer retaining control over improving task performance which might recommend to customize autonomy (Gopinath et al., 2017). Additionally, intuitive feedback to the user might be a key to yield transparent behavior of semi-autonomous control methods. This is supported by the conclusion that appropriate feedback could yield better user experience (Castellini et al., 2014).

While being less explored, afferent feedback to the user appears to be psychologically crucial for embodiment since it relies on multisensory integration of vision, touch, and proprioception (Giummarra et al., 2008; Christ et al., 2012). In addition to haptic feedback, Castellini et al. discuss closing the human–machine control loop by augmented reality techniques. While this seems interesting from a research and development perspective, feedback that integrates closer with the user might be perceived more natural and intuitive. Therefore, tactile feedback is promising, especially if the corresponding perceptual channels at the stump can be identified to induce referred sensations (Ehrsson et al., 2009). Kinesthetic feedback adjusting stiffness, or more general mechanical impedance behavior, might help to yield transparency and thereby create intuitive control (Jones and Hunter, 1990; Calanca and Fiorini, 2014; Castellini et al., 2014).

In conclusion, human-oriented approaches should be researched to enable considering users’ experiences and assessment in device and control design (Beckerle et al., 2017b). Castellini et al. (2014) recommended iterative user-centered design to improve the usability of robotic prostheses and to react to individual characteristics. Yet, taking human factors into account systematically and throughout the whole design process can help to spare iterations. Therefore, specific design methods and corresponding human factor models that generalize user requirements need to be developed (Beckerle et al., 2017a). Additionally, human-oriented measures based on user studies are necessary to evaluate the outcome (Castellini et al., 2015; Beckerle et al., 2017b). Appropriate assessment protocols should observe functionality from biomechanical and psychological perspectives in everyday tasks (Castellini et al., 2015). Beyond that, novel concepts for mirror therapy might draw on recent human-in-the-loop experimental designs that use robotic limb devices to investigate embodiment (Beckerle et al., 2016).

## Author Contributions

The author confirms being the sole contributor of this work and approved it for publication.

## Conflict of Interest Statement

The author declares that the research was conducted in the absence of any commercial or financial relationships that could be construed as a potential conflict of interest.
